# Admission to the intensive care unit: the need to study complexity and solutions

**DOI:** 10.1186/s13613-019-0494-y

**Published:** 2019-01-22

**Authors:** Ashraf Roshdy

**Affiliations:** 1 General Intensive Care Unit, St George’s University Hospitals NHS Foundation Trust, Blackshaw Road, Tooting, London, SW17 0QT UK; 2 Critical Care Department, Alexandria University, Alexandria, Egypt

Dear Editor,

Vulnerable patients are increasingly referred to intensive care units (ICU) reflecting population’s aging as well as long survival with chronic organ dysfunction and malignancies. The immense pressure on ICUs is expected to rise and hence the need to focus more research on ICU admission [1]. In this context comes the importance of Escher et al. study about the mortality prediction for patients referred to ICU [2]. Nevertheless, some comments are warranted for better understanding the subject complexity.

Classically, intensivists admit patients if they would benefit from, tolerate and agree ICU support. However, an increasingly limiting factor is the financial pressure on healthcare systems. Escher et al. defined benefit in term of 28-day survival despite more than half of their enrolled patients had advanced diseases [2]. For this particular group, other end points can be more relevant: long-term survival (e.g., 1 year), post-ICU morbidities and quality of life. One particular useful marker can be the quality adjusted life year (QALY), especially when combined with cost calculation in form of incremental cost per QALY (ICER) gained in ICU versus the ward [3].

Ethical issues are fundamental when studying ICU admission [4]. In many instances, declining admission ends by patient’s deterioration and may be death. Quality of life also lacks a clear definition which can differ significantly between healthcare payers, providers and patients [4]. Moreover, the accepted ICER can vary from community to another and according to the way healthcare is financed (public, insurance based or private). As such, a transparent discussion gathering all stakeholders is needed in order to rationalize admission policies.

ICUs are heterogenous in terms of patients, structure, protocols, tools, but also doctors. Intensivists differ in background, training, subspecialty and experience; it may be of interest studying admission decision agreement among intensivists. Moreover, prognostication is affected by treatment withholding and withdrawal. It is obvious that intensivists’ prediction can be translated at some point to one of those limits, affecting the results’ validity. As such, it would be of benefit to include a clear policy in the protocol. It is not surprising too that guidelines and scores were practically of little help while national or local studies can be of better impact than international multicentric ones in this topic.

Thinking forward can be by re-structuring hospitals. Half of the patients in Escher et al. study were referred for respiratory support. Applying high flow nasal oxygen and noninvasive ventilation in acute wards or intermediate care units supported by outreach ICU team is both feasible and pragmatic. Trial admission also had been suggested [5]. It can be of interest comparing survival and cost between such settings and ICU.

Last, it was alarming how much physicians were confident in their decision. In contrast, within the highest predicted mortality group (> 90%) by intensivists, a third survived. While physicians in acute situations are trained to be decisive and confident, continuous reflection and reassessment reflect good medical practice. Intensive care medicine is a dynamic specialty where close monitoring and decision revision are cornerstone. It is not of harm to keep some internal hesitancy.

To sum up, Escher et al. work added an important piece to the puzzle, but there is a need for more studies exploring the complexity surrounding admission to the ICU. Admission principles interplay and cannot be studied separately. Early referral and discussion, documentation of patients’ preferences, trial admission, treatment escalation plans, step-up areas within the wards and most importantly public involvement and awareness can be the way forward.
